# “Not only standing for, but standing up for diversity”: an intersectional qualitative analysis of how university students embody and resist discrimination

**DOI:** 10.1186/s12939-026-03003-w

**Published:** 2026-09-05

**Authors:** Laura Pilz González, Christina Prediger, Christiane Stock, Hürrem Tezcan-Güntekin, Katherina Heinrichs

**Affiliations:** 1 Charité – University Medical Center Berlin, Corporate Member of Freie Universität Berlin and Humboldt-Universität zu Berlin, Institute of Health and Nursing Science, Augustenburger Platz 1, 13353 Berlin, Germany; 2 University of Southern Denmark, Unit for Health Promotion Research, Degnevej 14, Esbjerg, 6705 Denmark; 3 Alice Salomon University of Applied Science, Department of Health and Education, Berlin, Germany

**Keywords:** Discrimination, Mental health, Social inequity, Intersectionality, Higher education, Students

## Abstract

**Background:**

Universities are key social institutions that shape student health, wellbeing, and future trajectories. However, they are also embedded in, and contribute to, existing social inequities. Students holding marginalised positions consistently report experiences of discrimination within higher education. Such experiences adversely affect their mental health, sense of belonging, and academic trajectories. Much of this research relies on single-axis analyses, failing to capture the complexity of discrimination. Therefore, this study takes an intersectional, qualitative multi-level approach to explore how university students in Germany experience discrimination during their studies and how this shapes their mental health and wellbeing.

**Methods:**

Fifteen semi-structured, problem-centred interviews were conducted with university students who reported experiencing discrimination. Data were qualitatively analysed using the Intersectional Multilevel Analysis (IMA) framework by Winker and Degele, linking individual meaning-making and self-positioning to symbolic representations and social structures. Our approach combined inductive reconstruction of students’ self-positionings with deductive contextualisation of how discrimination operates across interpersonal, institutional, and structural levels.

**Results:**

Students experienced discrimination as an embodied process shaped by microaggressions, institutional rigidity, and unequal power relations – particularly within student–lecturer dynamics. These dynamics involved intersecting forms of inequity, including racism, sexism, xenophobia, classism, language-based exclusion, ableism, caregiving discrimination, and ageism, experienced not as isolated categories but as overlapping, context-dependent processes. Participants described how the experienced discrimination settled into the body through emotional, cognitive, and physical manifestations expressed as fear, anger, exhaustion, and burnout, accompanied by existential questioning and disrupted identities. These embodied effects were not isolated reactions but ongoing responses to inequity. Alongside these harms, students exercised agency through strategies of endurance, withdrawal and resistance as forms of embodied survival work in response to persistent inequities. Overall, discrimination emerged as a relational and institutional process involving fluid interpersonal dynamics and contextual negotiations through which students’ academic trajectories and relationships to power are continuously (re)produced.

**Conclusion:**

Discrimination in higher education must be recognised as not only a social issue, but also a critical public health concern determining students' mental health and wellbeing. Addressing its embodied consequences demands institutional accountability and policies that move beyond symbolic diversity rhetoric toward meaningful structural and cultural transformation.

**Supplementary Information:**

The online version contains supplementary material available at https://doi.org/10.1186/s12939-026-03003-w.

## Abstract in German (original language)

**Hintergrund:**

Hochschulen sind bedeutsame soziale Einrichtungen, die Einfluss auf Gesundheit, Wohlbefinden und Zukunftsaussichten Studierender nehmen. Gleichzeitig sind sie jedoch auch Teil bestehender sozialer Ungleichheiten und tragen zu diesen bei. Studierende, die sich in einer marginalisierten Position befinden, berichten häufiger als andere von Diskriminierungserfahrungen. Solche Erfahrungen wirken sich negativ auf ihre psychische Gesundheit, ihr Zugehörigkeitsgefühl und ihren akademischen Werdegang aus. Ein Großteil der bisherigen Forschung greift auf eindimensionale Analyseansätze zurück und verfehlt somit, die Komplexität von Diskriminierungserfahrungen adäquat abzubilden. Diese Studie verfolgt daher einen intersektionalen, qualitativen Mehrebenenansatz und untersucht, wie Studierende an deutschen Hochschulen während ihres Studiums Diskriminierung erfahren und wie sich dies auf ihre psychische Gesundheit und ihr Wohlbefinden auswirkt.

**Methoden:**

Fünfzehn problemzentrierte, semi-strukturierte Interviews wurden mit Studierenden durchgeführt, die während des Studiums Diskriminierung erlebt haben. Die qualitative Auswertung erfolgte auf Grundlage der Intersektionalen Mehrebenenanalyse (IMA) von Winker und Degele, welche individuelle Bedeutungszuschreibungen und Positionierungen mit symbolischen Repräsentationen und sozialen Strukturen verknüpft. Unsere Analyse kombinierte eine induktive Rekonstruktion der Selbstidentifikation der Teilnehmenden mit einer deduktiven Kontextualisierung von Diskriminierung auf interpersonaler, institutioneller und struktureller Ebene.

**Ergebnisse:**

Studierende erlebten Diskriminierung als einen leiblichen Prozess, der durch Mikroaggressionen, institutionelle Starrheit und asymmetrische Machtverhältnisse geprägt war – insbesondere in der Beziehung zu Lehrenden. Dabei wurden vielfältige, sich überschneidende Diskriminierungsformen sichtbar, darunter Rassismus, Sexismus, Xenophobie, Klassismus, sprachbezogener Ausschluss, Ableismus, Diskriminierung aufgrund von Sorgeverantwortung sowie Altersdiskriminierung. Die erlebte Diskriminierung manifestierte sich emotional, kognitiv und körperlich – in Form von Angst, Wut, Erschöpfung und Burnout, begleitet von Identitätsverunsicherungen. Diese Erfahrungen waren keine kurzfristigen Reaktionen, sondern langanhaltende Aushandlungsprozesse im Rahmen struktureller Ungleichheit. Gleichzeitig zu diesen Belastungen zeigten die Studierenden Handlungsfähigkeit in Form von Strategien des Durchhaltens, des Rückzugs oder des Widerstands als Ausprägungen der leiblichen Überlebensarbeit. Diese waren eine Reaktion auf anhaltende Ungleichheiten. Insgesamt zeigte sich Diskriminierung als relationaler und institutionell verankerter Prozess, der akademische Laufbahnen und Machtverhältnisse kontinuierlich (re)produziert.

**Schlussfolgerungen:**

Diskriminierung im Hochschulkontext ist nicht nur ein soziales Problem, sondern auch ein relevantes Thema der öffentlichen Gesundheit und beeinflusst die psychische Gesundheit sowie das Wohlbefinden Studierender. Um die damit verbundenen Folgen anzugehen, sind institutionelle Verantwortung und Maßnahmen erforderlich, die über symbolische Diversitätsrhetorik hinausgehen und tiefgreifende strukturelle und kulturelle Veränderungen anstreben.

## Background

Universities are key social institutions, shaping wellbeing, social mobility, and future opportunities. At the same time, they are also embedded in, and contribute to, broader structures of inequity. Consequently, they sustain and create hierarchies based on race, gender, migration background, (dis)ability, socioeconomic status, and other socio-constructed positions [1, 2]. Research consistently shows that students holding marginalised positions along such axes often encounter discrimination within higher education [2–5]. Discrimination is understood here as the unequal or prejudicial treatment of individuals or groups on the basis of socially prescribed and constructed characteristics. Rather than merely a subjective experience, discrimination is a structural mechanism that contributes to social inequalities across diverse populations [6, 7]. However, much of this research continues to examine discrimination through isolated or additive social categories, limiting our understanding of how discrimination is enacted through intersecting structures of power.

To address this gap, we draw on intersectionality, a framework coined by Crenshaw [8] and rooted in Black feminist thought and activism [9]. Intersectionality emerged in response to single-axis analyses failing to capture the complexity of lived oppression [10, 11], revealing how race, gender, class, and other socio-constructed positions interact to produce distinct forms of exclusion [10]. It shifts the focus from who is marginalised to how institutions are structured to protect some and expose others to harm [12, 13]. When applied to higher education, this perspective urges us to examine how institutional policies, social norms, and interpersonal dynamics generate unequal exposure to harm and how those most impacted have to navigate these harms without structural protection. At the same time, intersectionality demands that we centre the experiences of those affected as agents of knowledge, survival, and resistance rather than as mere objects of study [10].

Building on this framework, we approach the *norm* not merely as a demographic majority, but as a system of institutional power that has been constructed over time [14]. Drawing on Black feminist and decolonial thought, we conceptualise institutional normativity as being anchored in *whiteness*, not as a racial category, but as an epistemic and structural position that defines what is considered credible, neutral, or valuable within, in our case, academic settings [15–17]. Institutional whiteness thus establishes the status quo against which legitimacy is measured, systematically marginalising bodies, knowledge, and ways of being that fall outside its frame [14, 15]. This process, often described as *othering*, marks certain groups as different, deficient, or out of place in relation to dominant norms [15, 18] and thereby creates and reinforces social boundaries and hierarchies.

Far from being a neutral standard, institutional whiteness manifests as exclusion, silencing, and devaluation in everyday experiences, resulting in unequal treatment and discrimination [15, 19]. From a public health perspective, the minority stress model helps to explain how university students who experience discrimination, whether directly, in anticipation, or by witnessing it, face cumulative psychological burdens [20, 21]. The consequences comprise stress, anxiety, depressive symptoms, hypervigilance, and social withdrawal [7, 22]. Even without direct targeting, a climate of exclusion and a persistent fear of harm can erode students’ psychological safety and sense of belonging, particularly among already marginalised groups [22, 23]. Discrimination can operate through everyday interactions and tacit norms, as well as through explicit acts or policies [7, 22]. These influences shape what is perceived as possible, desirable, or risky, even in the absence of formal coercion. In response, students may adapt their behaviour and expectations in anticipation of unequal treatment, perpetuating cycles of self-censorship, disengagement, or resistance [15]. As such, discrimination within higher education should be considered a structural determinant of student mental health and wellbeing [24, 25].

Across contexts, the neo-liberalisation of diversity rhetoric in higher education has resulted in superficial, symbolic commitments to diversity [26–29]. In Germany for example, existing measures such as reporting structures are often unclear or unsafe, and institutional inaction leaves affected students without adequate protection [26]. As a result, the burden of recognising, enduring, and confronting inequity often falls on those most harmed [30], a phenomenon sometimes described as the *minority tax*, which captures the emotional, cognitive, and social costs of surviving in institutions not built for them [31].

While these dynamics are not unique to any one national context, their specific configurations vary. Germany presents a particularly relevant case, as despite theoretically having open access to university education, social mobility and access to university is closely associated with family background and social class [32]. A comparative report by the Organisation for Economic Co-operation and Development (OECD) highlights this long-standing inequality, showing that while it takes an average of 4.5 generations to move from the lowest to the middle income group across OECD countries, it takes six generations in Germany [33]. In addition, despite an increasingly diverse student population, the prevailing image of the “typical” student in Germany remains unchanged: under 30 years old, white, with a straightforward path from school to university and no care responsibilities or employment commitments [34]. Meanwhile, a nationwide survey reported that around 26% of university students had experienced discrimination during their studies, primarily on the basis of gender and non-German origin [35]. Further research indicates that discrimination among students is often perpetrated by lecturers and associated with poorer mental health outcomes for gender-diverse students, first-generation students, and other marginalised groups [36]. These findings underscore the association of discrimination with psychological distress, reduced academic attainment, and increased risk of dropping out [4, 37, 38].

In response, this study adopts an intersectional, qualitative, multi-level approach to explore how students experience discrimination, and how such experiences shape their mental health and wellbeing within the context of German higher education. By centring student narratives, we aim not only to understand the dynamics of discrimination but also to challenge dominant institutional framings, thereby, contributing to more accountable and justice-oriented approaches to equity in academia.

## Methods

This study draws on fifteen problem-centred, semi-structured interviews [39] exploring the lived experiences of discrimination in higher education among university students. Rather than predefining marginalised categories, we centred participants’ experiences of inequity and their self-positioning [10], aiming to foreground how students understand, navigate, and experience discrimination as socially situated practices.

Our analytical approach was guided by Winker and Degele’s [40] Intersectional Multi-Level Analysis (IMA) which conceptualises intersectionality as the interaction between symbolic representations, institutional structures, and individual meaning-making. Through the subject-oriented and intersectional lens of the IMA [40, 41], we conceptualised students’ experiences and responses not as individual coping deficits but as strategies of resistance, survival, and meaning-making in the context of inequity in higher education.

As an empirical method, the IMA enabled us to reconstruct the relationship between structural constraints and agency, by linking individual meaning-making and self-positioning to broader structural and symbolic dimensions, and tracing how discrimination operates across interpersonal, institutional, and structural levels. As in all qualitative research, our beliefs, experiences, and interests influenced our decision-making and actions, informed and shaped the research process.

In reporting this study, we followed the Big Q Reporting Guidelines [42] wherever possible. Accordingly, we report the characteristics of the participants in the methods section.

### Participants and participant involvement

Students responded to study information materials and chose to participate based on their own experiences of discrimination. This approach resulted in a convenience sample [40]. As discrimination in higher education can manifest across multiple intersecting dimensions, participation was based on students’ own accounts of having experienced or currently experiencing discrimination during their studies.

Information about the study was disseminated through student-led organisations and university diversity and inclusion offices across several universities in a large German city. Flyers in German and English were made available in libraries, cafeterias, and other student spaces to encourage interest. Students who wished to participate contacted the first author (LPG) via email, after which they received written study information and consent materials at least 24 h prior to scheduling an interview. Before interviews began, the participants had given written informed consent. Participants could choose whether they wanted to take part in the interviews in person or online via Microsoft Teams, and in either English or German.

Given our subject-oriented and intersectional approach, we did not rely on data saturation to determine the size of the sample, since saturation assumes relatively stable phenomena and homogeneous meanings across cases [43]. Instead, we followed the concept of information power [43], which suggests that smaller samples may be sufficient when interviews yield rich, relevant, and in-depth information in relation to the study aim. Accordingly, we continuously assessed the quality of the dialogue and the analytical depth of the material in relation to the research questions. This approach aligns with IMA-specific methodological guidance, which emphasises that scope and depth depend on the research context, timeframe, and analytical ambition rather than fixed sample sizes [41].

In keeping with IMA guidance, sociodemographic information was collected only after each interview through a questionnaire [41] based on the Diversity Minimal Item Set [44], as a guiding framework to address the diversity and gender gap within health research. This allowed us to subsequently examine whether and how participants positioned themselves in relation to social categories when constructing and interpreting their experiences. Beyond this orienting and contextual function, sociodemographic characteristics were not used as predefined analytical variables or explanatory categories in the interpretation process. Accordingly, social difference categories were approached as emergent, relational, and context-dependent rather than as fixed axes of comparison [10]. To support contextual interpretation and enhance transferability, sociodemographic characteristics are reported descriptively (see Table 1), while cautioning against reading these categories as fixed, essential, independent, or causal.

**Table 1:** Participants’ characteristics (*n* = 15)

Participant characteristic	n or mean (range)
**Demographics**	
Gender (self-identified)^1^	
Female	9
Male	6
Sexual orientation (self-identified)	
Heterosexual	13
Gay/lesbian	1
Pansexual	1
Age in years	26.6 (19–55)
Ethnic minority (self-identified)	
Yes	7
No	8
Migration background^2^	
None	5
Mixed	3
Own migration	6
No answer	1
First language	
German only	6
German + other	2
Non-German	6
No answer	1
Religion	
None	7
Christian	4
Muslim	4
**Health (self-reported)**	
(Dis)ability	
Yes	4
No	11
Physical chronic health condition	
Yes	5
No	9
No answer	1
Mental health condition	
Yes	5
No	10
Neurodiversity	
Yes	2
No	13
**Education and study context**	
Study programme	
Bachelor	2
Master	4
State exam^3^	8
PhD	1
Study field	
Medicine	8
Public health/health sciences	3
Education/social sciences	3
Humanities	1
First-generation academic	
Yes	4
No	11
**Socioeconomic and care responsibilities**	
Employment next to studies	
Yes	11
No	4
Care work next to studies	
Yes	6
No	9
**Interview conditions**	
Language of interview	
German	13
English	2
Interview mode	
In person	8
Online	5
Interview duration in hours: minutes	00:52 (00:35–01:22)

Notes: ^1^Gender: participants could select multiple and non-binary gender identities. All participants identified exclusively as female or male. ^2^Migration background: none = participant and both parents born in the study country; mixed = participant born in study country, parents born in different countries (including cases where one parent was born in the study country); own migration = participant born abroad. ^3^German qualifying degree (e.g., in medicine)

### Ethics

Prior to data collection, all participants gave informed consent. To ensure confidentiality and anonymity, all potentially identifying information was removed from the transcripts, and participants are referred to using randomly assigned three-digit numerical identification codes. This study was performed in line with the principles of the Declaration of Helsinki. Approval was granted by the Ethics Committee of Charité – Universitätsmedizin Berlin (date: 29.10.2024/No. EA1/237/24).

Participants received a monetary compensation (30€) in recognition of their time, expertise, and emotional labour. The amount was set to acknowledge participation as legitimate work. Transcription was conducted by a professional provider under a data protection agreement, partially funded through a project grant from Techniker Krankenkasse to support the development of a student health service.

### Interviews

A problem-centred, semi-structured interview guide [39] was developed by LPG, informed by intersectional perspectives and prior research on discrimination and power, and discussed with the second (CP) and last authors (KH). This form of interview design was chosen as it facilitates an open, narrative-driven exploration of lived experience while directing attention towards a defined social problem [39], in our case, discrimination in higher education. The interviews began with open questions about identity and self-positioning, followed by prompts about situations of perceived discrimination at university, the perceived emotional and academic impacts of this discrimination, and the strategies used to navigate these conditions. Participants were also invited to reflect on institutional factors, accountability mechanisms and possibilities for improvement. The guide was used flexibly to allow emergent themes to shape the conversation (see interview guide in appendix A).

The interviews were conducted by LPG between December 2024 and March 2025 and ranged in length from 35 min to 1 h and 22 min, with an average duration of 52 min. None of the interviewees were known to the researcher before data collection. All interviews were audio-recorded and transcribed verbatim by a professional transcription service that was bound by a data protection agreement. Field notes in the form of memos were recorded during or after each interview to capture contextual impressions [41, 45]. LPG pseudonymised the transcripts and cross-checked them against the audio recordings for accuracy; the audio files were subsequently deleted. No linguistic corrections were applied during this process speech patterns and grammatical inconsistencies were retained to preserve the authenticity of the participants. This means that some quotes may contain informal or non-standard speech, which we chose not to edit or mark as “errors.” The interviews were analysed in their original language. Only the German quotes presented here were translated into English by LPG and KH; quotes originally spoken in English appear as they were.

### Data analysis

We analysed the interviews using the IMA, guided by Ganz and Hausotter’s [41] step-by-step procedure. The analysis proceeded in two phases, comprising eight steps (A–H). Analytically, the IMA focuses on three levels of interaction: (1) subject constructions (self-positioning and meaning making); (2) symbolic representations (e.g. norms, values, stereotypes, and dominant discourses); and (3) social structures (e.g. institutional arrangements, policies, and hierarchical relations).

In the first phase (steps A-D), each interview was analysed inductively to reconstruct participants’ self-positionings in relation to the three levels. Rather than assuming the relevance of specific categories, our analysis examined how participants positioned themselves when accounting for discriminatory experiences. This analysis was summarised in short identity constructions for each participant. The process included the following steps: (A) LPG developed identity constructions for each interview. (B) Three of these constructions were developed collaboratively by the LPG and the second author (CP), first independently and then merged to surface any interpretive assumptions and ensure close grounding in the data. (C) The last author (KH) independently read and commented on these reconstructions, providing additional perspectives and challenging potential blind spots. In keeping with a reflexive qualitative paradigm, these procedures were not undertaken to achieve coder agreement, but rather to strengthen interpretive rigour through critical dialogue, theoretical sensitivity, and analytic transparency [46]. (D) Finally, the identity constructions were returned to the participants for communicative validation [41]. All participants were contacted for communicative validation; two suggested minor clarifications that did not affect the overall analysis.

The second analytical phase (steps E-H), shifted from individual narratives to collective patterns across the dataset. E) The material was reread and analysed in relation to structural power dynamics, including e.g. classisms, heteronormativism, racisms, and bodyisms [40], enabling the identification of implicit, invisible, or unspoken inequities [41]. While theoretically informed and deductively applied, these categories were treated as relational, intersectional, and context-dependent rather than fixed or essential [41]. This approach prevented the reification of inequities while enabling structural forces to become empirically visible. F) Initial analytical impressions were discussed with CP, who had already conducted the familiarisation process with three interviews. In the role of a “critical friend” [46], CP provided constructive feedback to support reflexive engagement; KH supervised this process. Throughout the analysis, agency was understood as the situated capacity to act within structurally constrained conditions [47], foregrounding how students navigate, negotiate, and contest discrimination rather than framing their responses as individual coping deficits or symptoms. G) We then deepened the analysis by examining how participants made sense of, justified, contested, or at times (re)produced institutionalised inequities. This step illuminated how students engaged with dominant norms, and where moments of critique or accommodation emerged. H) Finally, we synthesised the findings across all three analytical levels, generating a comprehensive account of how discrimination was experienced. To further enhance interpretive transparency of this step, the preliminary results were discussed with the fourth author (HTG).

Combining inductive reconstruction with deductive contextualisation enabled a nuanced analysis of how discrimination is shaped and experienced in higher education and how students exercise agency within these constraints. The concept of *embodiment*, which views the body as a site where social inequalities and power imbalances – such as those related to discrimination – materialise and are expressed physically and emotionally [48, 49], emerged inductively rather than being a predefined theoretical framework.

### Researcher reflexivity and own positionality

In line with reflexive qualitative inquiry, we recognise that knowledge is co-constructed and shaped by the positionalities of researchers. The first author (LPG) identifies as a cisgender woman with a Latinx-European migration background, whose first language is not German and who has personal experience with discrimination in higher education. These experiences provided sensitivity towards institutional barriers, emotional labour, and subtle forms of exclusion, while also requiring ongoing reflexive attention to avoid over-identifying with participants. At the same time, LPG occupies positions of privilege (e.g., educational status, citizenship, and whiteness in the German context), which facilitated institutional access and influenced how participants positioned themselves during interviews. Regular memo-writing and reflexive dialogue were employed to critically interrogate interpretive assumptions.

All co-authors occupy more structurally privileged positions regarding employment security, academic seniority, and socioeconomic status. Their more distanced perspectives, combined with their professional expertise in social work, gender studies, social pedagogy, psychology, public health, and qualitative methods, helped to question moments of potential resonance-based bias.

We acknowledge that our beliefs, experiences, and interests influenced our decision-making and actions, thereby shaping the research.

## Results

Our analysis showed that students’ experiences of discrimination were not isolated events, but part of a dynamic process shaped by intersecting power relations across interpersonal, institutional, and structural levels. Three interrelated themes captured this process: (1) encountering and interpreting discrimination: from uncertainty to recognition, (2) embodiment and situated navigation of discrimination, and (3) restructuring and (re)production of power: negotiating frontlines. Together, these themes illustrate how discrimination was encountered, embodied, navigated, and variously (re)produced or contested within higher education.

### Encountering and interpreting discrimination: from uncertainty to recognition

#### Uncertainty, doubt, and the gradual naming of discrimination

Students rarely recognised discriminatory experiences immediately. Instead, such encounters often began with uncertainty, ambiguity, or self-questioning. Many hesitated to label incidents as discriminatory, downplaying or comparing their own experiences to *“others who may have it worse” (140, Pos. 11)* or questioning their interpretations.

Subtle forms of exclusion, such as patronising behaviour or the invisibility of non-normative identities, frequently prompted self-doubt. As a result, students asked themselves if they were the ones in the wrong – or their fellow students or their lecturers. The recognition of discrimination often emerged retrospectively, triggered by personal reflection or conversations with trusted others.

For me, it came afterwards. At first, I just thought, […] ‘Well, he [the examinator] said it [discriminatory comment],’ and it went in one ear and out the other. […] Two days after the exam I told my dad, and he said, ‘That shouldn’t be.’ And that I should report this […]. In the moment I genuinely hadn’t seen it [as discrimination]. It was only later that I realised it was something I could act on and that the hurt I felt wasn’t hypersensitivity, but genuine discrimination that can be labelled as such.” (470, Pos. 45)

For some, even being able to talk about their experiences in the interview was a relief; for others, the interview itself was perceived as a therapeutic process, enabling them to articulate what had previously been kept silent.

#### Intersecting forms of othering

Across their narratives, students described racialised, gendered, socio-economic, linguistic, religious, bodily, and life-course-related dimensions along which they experienced discrimination. These social positions shaped the specific forms of unequal treatment they encountered, including racism, xenophobia, language-based exclusion, anti-Muslim racism, sexism and sexual harassment, classism, ableism, ageism, and discrimination tied to pregnancy and caregiving.

These forms of discrimination rarely appeared as isolated social categories, but rather as intersecting, context-dependent processes that marked certain bodies, life realities, or identities as “*falling out of the pattern”* (619, Pos. 124) and not fitting in within academic spaces.

*The role of being a woman (laughs)*,* first a woman with a migration background*,* followed by being a mother*,* then a student. These are different intersectional issues*,* and we are groups of people who are extremely affected by disadvantage. And I recognise this role*,* and I understand my role more now. And I know that my identity is not constructed by disadvantage*,* but as a person who is trying to live beyond that disadvantage.* (859, Pos. 15)

#### Sources and contexts of discrimination

The interpretive process of discrimination was shaped by the context in which it occurred. Lecturers were commonly identified as central actors. Students recounted dismissive comments, microaggressions, biased grading, and value-laden remarks about language, bodies, religion, or background. Institutional rigidity in exams, attendance policies, and curricula further reinforced disadvantage. Students emphasised that institutional silence and peer inaction intensified the harm and hurt the most.

*What hurt me most afterwards is the complete lack of reaction from my fellow students. No one joined the discussion or spoke up to say*,* ‘That’s not okay.’ […] One classmate did come to me afterwards and said she wasn’t okay with that*,* but in the moment*,* no one [said anything]. (495*,* Pos. 15)*

Additionally, broader socio-political climates – including rising right-wing narratives in Germany, public debates about migration, racism, and religion, as well as entrenched majoritarian academic norms –shaped how students anticipated and interpreted discriminatory events, intensifying fears about safety, visibility, and future opportunities.

*I feel fear about what’s happening in the country*,* and because you don’t feel that sense of security at university anymore*,* that fear from the broader social situation spills over into the seminar room. (531*,* Pos. 71)*

These accounts illustrate how encountering and naming discrimination was not simply an event but the initial step in navigating discrimination shaped by unequal power relations and shifts in the political contexts.

### Embodiment and situated navigation of inequity

#### Embodiment of inequity: how inequity becomes lived and felt

Participants’ narratives showed that, once experienced, discrimination was perceived as an embodied sensation that accompanied them over time and was not limited to a single event. These embodied experiences were lived through emotional, cognitive, and physical processes. Invalidation, humiliation, and hostility manifested as emotional pain, shame, anger, and loneliness, as well as persistent tension, fatigue, and exhaustion. Many students spoke of rumination or burnout, noting how discriminatory environments drained their energy and motivation: “*Discrimination makes you tired. Being discriminated*,* having to prove yourself*,* is very exhausting.”* (859, Pos. 65).

Some students experienced chronic worry or hypervigilance, especially in interactions with lecturers, seminar groups, or before/during examinations. The university was then often described as a place that felt unsafe or where they constantly had to be on guard anticipating discrimination, while getting back home represented the only space where they could “exhale” and let their guard down.

*I often find it really stressful because I know there are so many factors I can’t influence. I mean*,* I’m already stressed because I’m studying for an exam*,* and then I have to think about*,* whether I should wear a black headscarf. […] And if I show up in my black dress and also a black headscarf*,* maybe I’ll just wear a blue one for the exam*,* even though I actually prefer wearing the black one*,* you know? Those are just things I have to pay attention to. […] Should I tell the person [the examinator] that I have an illness? Will they end up thinking: ‘You didn’t have to tell me that?’ […] For others maybe: ‘Okay*,* I’ll simply go to the exam.’ And for me*,* it’s also a question of*,* okay*,* how do I go there*,* how do I speak? Because those are the kinds of things that make people pay even more attention to the way I speak. (387*,* Pos. 53)*

#### Navigating unequal environments: situated agency

At the same time, navigating power relations and structures was not just a source of harm; it also became a space for situated agency. Students described a range of strategies to navigate and contest discriminatory environments, sometimes implicitly and sometimes deliberately. They often adapted their behaviour in anticipation of unequal treatment. Some distanced themselves emotionally, reframed experiences as more manageable, or normalised certain harms as a means of preserving day-to-day functioning. One participant even mentioned dissociating so strongly that a protective alter ego emerged to buffer harm.

*I have to say*,* when I’m in a professional setting or at university*,* I’m not myself. I have an alter ego*,* [the participant explicitly gave the alter ego the own first name followed by “Two”]*,* who lives at the hospital and the university and does his thing there. And whatever happens to him*,* I don’t take home with me. As long as the everyday or clinical routine runs smoothly*,* there’s no need to get worked up; you could just stay factual. […] [Own name]-Two has more patience*,* and maybe because of that*,* the pain threshold is a bit higher.* (725, Pos. 67)

Others managed their visibility carefully by concealing aspects of their identity, such as their accent, appearance and/or chronic disease, monitoring how they presented themselves and adjusting their expectations of fairness in environments where power imbalances were particularly pronounced. Focusing on personal anchors outside of university, such as sports, family and friends, or work, as well as counter-narratives or the hope that things will get better also helped students to navigate discriminatory environments.

#### Collective forms of situated agency

Alongside individual strategies, students relied heavily on relational and collective forms of situated agency. Peer groups provided validation, a shared understanding and a sense of safety. For many, solidarity with other marginalised students was crucial in helping them endure the emotional toll of discrimination alongside their academic studies. While such spaces provided emotional support and mutual care, they also represent the segregating effects of discrimination.

*I’d say I had more contact with students with a migration background […] and those with recent migration experience. […] We were always together and*,* in a way*,* identified as a group. We also tended to form group work together. And I’m wondering whether we formed this group work because we were afraid the others wouldn’t choose us or because we genuinely got along well. I’m asking myself whether it was because we liked each other or because we had similar problems. […] We did have similar problems. And maybe that’s why we were afraid of being discriminated by the others*,* and so we worked together. That’s what I’m wondering.* (859, Pos. 89)

Collective engagement also held political significance, as for some students, solidarity served as an implicit challenge to institutional neglect, creating small pockets of resistance within an otherwise inequitable environment. However, even these spaces were shaped by power dynamics and dominant norms, which determined which struggles and identities were recognised within academic environments. In reflecting on campus discussions surrounding the Israel–Palestine conflict, some students described frustration with what they perceived as selective institutional neutrality or implicitly aligned with dominant political narratives. Calls to remain apolitical were experienced as silencing conversations about racism and reinforcing the sense that issues affecting racialised and other marginalised communities were considered peripheral to the university’s concerns.

*And then*,* there it was again*,* ‘What does that [Israel-Palestina conflict] have to do with us? Why should we comment on it?’ It was a reminder that topics like racism*,* or anything affecting non-white people*,* have no real place in the discourse. That brought up a lot of mistrust and frustration towards my white fellow students. I felt that sense of mistrust and the fear of not really belonging. (495*, *Pos. 43)*

### Restructured and (re)produced relations of power: negotiating frontlines

Students’ experiences of discrimination did not end with individual encounters but accumulated into a broader restructuring of how they understood themselves, their relationships, and their futures within and beyond university. This theme captures how discrimination operated simultaneously at interpersonal, institutional, and structural levels, and how these forces became internalised shaping students’ dispositions, expectations, and trajectories. Over time, the interplay between embodiment and situated agency crystallised into three trajectories within unequal environments: endurance, withdrawal, and resistance.

#### Endurance: staying in the system at personal cost

Many students described how they kept going within the system while absorbing its demands. Unequal power relations, particularly within the student-lecturer relationship, created lasting feelings of vulnerability, insecurity, and conditional belonging. Challenging discriminatory behaviour felt too risky because lecturers held significant control over grading, progression, and reputation. Students described finding themselves caught between self-silencing to avoid repercussions and feeling guilty for not speaking out.

*I feel regretful that I didn’t stand up for myself*,* but also powerless when I relive it. I don’t think I could have done anything differently*,* either risk making a bad name for myself in front of the professors on my first week of study or continue to compromise and continue to let it affect me and my studies and like my stress around my studies. […] I’d just moved abroad; I’m an international student starting a competitive master’s programme. (258*,* Pos. 35)*

Endurance often involved studying out of fear – more hours, less effectively due to the emotional pressure – driven by a *“survival drive” (470, Pos. 49)* rather than by curiosity or interest. Students felt the need to continually prove themselves or overperform to fit in and to accept hypervigilance as part of academic life, including being positioned as representatives of others who shared their marginalised identities.

#### Withdrawal: stepping back or exiting the system

Repeated marginalisation eroded motivation and self-efficacy, leading to different forms of withdrawal from unequal systems. At the social level, students avoided spaces dominated by whiteness or peers they did not feel safe around. At the academic level, students showed withdrawal in the form of disengagement or questioning their legitimacy in the programme. For some, withdrawal extended into a mental “exit plan” as an ongoing strategy for navigating discrimination by anticipating departure from the institution or even the country.

*And that’s why I have an exit plan*,* I always have this idea in my head of ‘just one more year’ or ‘just two more years*,*’ which somehow makes it easier to cope with things like the upcoming elections*,* racism at university*,* et cetera. And I think I’ve also just lowered my expectations of my white fellow students.* (495, Pos. 67)

Institutional and structural practices exacerbated withdrawal. Many students experienced a discrepancy between their universities’ public commitments to diversity and their everyday encounters with institutional indifference. Discriminatory lecturers continued to teach, reporting channels yielded little response, and resources were often lacking, unknown, or perceived as not representing them or their needs.

*Well*,* I think the measures are that there are no measures against discrimination. Because you can report it and everything*,* but after having reported it*,* what happened then? I still saw his name [lecturer who sexually harassed a student] on the website. So*,* he’s still teaching. (782*,* Pos. 55)*

Several students described the university as a place where they felt small, anonymous, or irrelevant, which reinforced the internalisation of marginality. These conditions normalised discrimination and fostered mistrust, shaping not only daily functioning but also long-term educational and career decisions.

Broader socio-political climate and prior educational experiences also contributed to withdrawal. Participants referred to the institutional whiteness of academic expectations, as well as to structural inequities in the German school system. Together, these factors created an implicit template of who “belongs” in university spaces. Discriminatory encounters early on in school had already restricted access to preferred programmes of study or shaped expectations regarding scholarships and academic careers, even at PhD level.

*If you look at the educational system at school in Germany*,* this four-track system where pupils are divided up after fourth grade*,* at the age of ten […]. It has a huge impact on self-confidence or determines so much of a person’s later life path. It has so much to do with self-confidence*,* with emotions*,* with everything*,* and with all forms of discrimination. […] These forms of societal discrimination have a very strong effect*,* also in childhood*,* during socialisation. And I would say it runs through your whole life. […] So*,* yes*,* in any case*,* it continues until university*,* or rather*,* it doesn’t even get to university because of that.* (931, Pos. 37)

These narratives demonstrate how discrimination becomes embedded across educational transitions, shaping long-term opportunities and forcing individuals to view withdrawal, whether temporary or permanent, as a means of survival within limited options.

#### Resistance: critique, solidarity, and reconfigured positionality

Alongside endurance and withdrawal, discriminatory experiences also fostered critique, solidarity, and more explicit forms of resistance, such as political awareness or activism. In addition, students reframed rest, self-care, and boundary-setting as quiet acts of resistance. These counter-practices did not erase structural disadvantages but reconfigured how power was lived and contested, illustrating that relations of power were both (re)produced and transformed. Through such acts, students reasserted dignity and belonging on their own terms, while refusing to absorb further harm, carry the minority tax of educating others or constantly overperform.

*So why do I have to be the one to point out to the university that there is racism*,* and racist professors and lectures? […] What about all the others who were in the lecture or all the left-leaning [academic discipline] students who don’t say anything?* (495, Pos. 75)

In this sense, acts of rest, self-care, and boundary-setting became subtle ways of reclaiming dignity and protecting wellbeing within constrained environments. Some students developed a strong commitment to challenge inequities within their future professions or to act as role models for others.

*I know that I’m a role model for many girls […]*,* because I didn’t have that as a young girl. I always saw women who wore a headscarf and thought*,* ‘She looks like my mum […]*,* my aunts.’ And I’m glad that I can now be that person on the other side*,* to embody that representation. […] But it comes at the cost of so many questions and comments. (140*,* Pos. 87)*

Encounters with empathetic lecturers or visible role models offered glimpses of alternative academic cultures and extended forms of agency, reinforcing students’ refusal to accept discrimination as inevitable. At the same time, several students described how speaking up or being politically active sometimes intensified their marginality, showing how complaint and resistance themselves are costly and can lead to further othering.

#### Negotiating frontlines

Taken together, endurance, withdrawal, and resistance illustrate how students continuously negotiate frontlines: weighing which struggles they can afford to fight, which they must endure, and when they need to step back to protect their wellbeing. In this way, the final stage of the process shows that discrimination does not merely impact wellbeing; it actively reconfigures belonging, self-perception, and imagined futures within and beyond higher education, while students’ interpretive practices, solidarities, and resistant forms of agency simultaneously (re)produce and, at times, transform relations of power. These restructurings then shape how students anticipate, re-encounter, and navigate future experiences of discrimination.

Discriminatory experiences not only reshaped students’ wellbeing and academic pathways, they also sharpened their vision of what universities should be. One participant expressed this critique and aspiration by asking how far institutions are willing to stand up for diversity through material accountability, taking responsibility for working towards “*a place where you never have to be afraid to stand up for your rights or to live as the person you are”* (470, Pos. 133).

## Discussion

This study explored how university students experience discrimination during their studies and how such experiences shape their mental health and wellbeing. Consistent with previous research, our findings highlight that discrimination in higher education is not a single, isolated event, but rather a multidimensional and relational process embedded within broader power structures [4, 50]. Students encounter context-dependent forms of othering that emphasise how institutional norms determine which bodies, behaviours, and life experiences are recognised as legitimate within higher education, and which are deemed “out of place”.

Our results show that the consequences of discrimination were neither immediate nor straightforward. Instead, they emerged as embodied experiences that accompanied students through emotional, cognitive, and physical processes. Consequently, participants reported cumulative distress, chronic hypervigilance, and emotional fatigue from constantly navigating unequal environments. While these findings align with minority stress theory [20, 21], they extend beyond psychological models by drawing on concepts of embodiment that view the body as a place where power relations become lived [48, 49]. Building on Ahmed’s [30] work on the cultural politics of emotion, we argue that students’ responses should not be interpreted merely as personal symptoms, but rather as social effects, namely, bodily expressions of structural violence and affective misrecognition.

Our results show that several students only recognised their experiences as discrimination after discussing them with peers or relatives, meaning that not all experiences of discrimination were immediately apparent. This further highlights Ahmed’s [50] critique of institutional silence, which delegitimises harm and shifts the burden of recognition and articulation onto those most affected. Expanding on this, our findings also resonate with Hernandez’s [51] work on racial unspeakability, showing that harm was often felt in the body before it could even be articulated. In this sense, institutions not only shape what can be said, but also what can be experienced, regulating both the expression and recognition of suffering [51]. Here, we find that embodiment again reveals the gap between what is socially acknowledged and what is silently endured.

By shifting the focus from individualised pathology to navigation as agency, our study contributes to critical health equity research that centres survival work, affective labour, and relational strategies. We integrate intersectionality, embodiment, and minority stress theory into a multilevel framework that captures both the structure and the substance of students’ experiences. Similar to Cornell and Kessi [52], our results show that students were not passive recipients of harm. Instead, they engaged in continuous interpretation, adaptation, and critique, even in the face of institutional indifference or hostility. These findings also resonate with Çolak et al.’s [53] work in the Belgian higher education context, which similarly highlights how racialised students navigate everyday forms of exclusion, misrecognition, and conditional belonging within university settings. In both studies, these experiences shaped not only students’ mental health and wellbeing, but also their very sense of self, their educational futures, and their perceived legitimacy within academic space [52, 53].

As participants repeatedly emphasised, harm was not only enacted through interpersonal conflict or overt hostility, but through the structural conditions of higher education itself. Here, our findings also resonate with those of Çolak et al. [54], who demonstrate how experiences of discrimination can be reproduced through everyday institutional practices despite universities’ commitments to equity and inclusion. Universities that position themselves as inclusive must confront the fact that harm is not always a breach of norms but often produced by the norms themselves. In this sense, our findings support existing critiques of diversity and inclusion strategies that focus primarily on symbolic/tokenistic representation or awareness-raising, without addressing the deeper structural conditions that produce harm [15, 26, 52]. As further research in higher education has highlighted, without working accountability mechanisms, safe – and effective – reporting systems, and a redistribution of responsibility away from those harmed, such measures risk (re)producing the very inequities they claim to address [26, 29, 55]. A way to move beyond symbolic/tokenistic measures is for universities to invest in independent, accessible, and protected reporting offices with the power to enforce consequences [29], thereby addressing one of the core institutional silences highlighted in our study.

Moving beyond critique by examining student agency through theories and methodologies from outside the mainstream of social psychology and public health, we were able to better understand how students exercise situated agency. In doing so, students also expressed visions of what universities should become. Rather than merely being inclusive in name, they should be institutions that are actively committed to structural accountability and recognition. Students also described the need for institutions where it is safe to acknowledge harm, to feel identified and to be recognised as individuals. These visions are not impractical, instead they are grounded in lived experience, solidarity, and critical insight. They represent what literature might call an affective orientation towards possibility, even under conditions of exhaustion and misrecognition [30].

Further literature underscores that such visions will remain unmet unless institutions confront and dismantle the very systems of power that determine who belongs, who is heard and who is protected. As Thompson and Zablotsky [29] argue, “rethinking” diversity in higher education must move beyond rhetoric toward enforceable institutional change. Organisational development initiatives, such as the two-year Diversity Audit “Shaping Diversity” implemented at scale in German higher education [56], may offer one potential mechanism. The audit functions as an externally guided organisational development process in which universities conduct self-assessments, develop institutional diversity strategies, and formulate action plans across multiple levels, with the aim of supporting their transformation into learning organisations [57]. Crucially, when organisational development processes are grounded in standpoint theory and implemented through participatory approaches, they hold the potential not only to diagnose institutional shortcomings but to catalyse deeper cultural transformation [52, 58].

To ensure sustainable change, faculty must move beyond diversity strategies that treat categories in isolation and, as Mergner et al. [59] frame it, adopt forms of *intersectional pedagogy*. In the context of higher education, this requires professional development formats (e.g. workshops), that translate critical theory into everyday teaching practice. These should support staff in acting as “change agents” by reflecting on their own positionality and critically dismantling standardised curricula that often marginalise non-dominant knowledge [59]. Staff and faculty, particularly those in supervisory or evaluative roles, should undergo compulsory training on power, discrimination, and structural inequity, grounded in lived experiences and critical frameworks, not just compliance checklists [59]. Moreover, equity indicators should be integrated into university evaluation and funding mechanisms, shifting incentives toward accountability and care rather than prestige alone [29, 55].

All in all, by grounding our study in an intersectional, institutional analysis such as the IMA, we re-emphasise that mental health outcomes are shaped not only by direct experiences of discrimination [22, 23], but also by the absence of collective care and institutional responsiveness. Structural change cannot merely be rhetorical; it must be tied to transparent tools, shifting responsibilities, and material consequences.

**Fig. 1:**
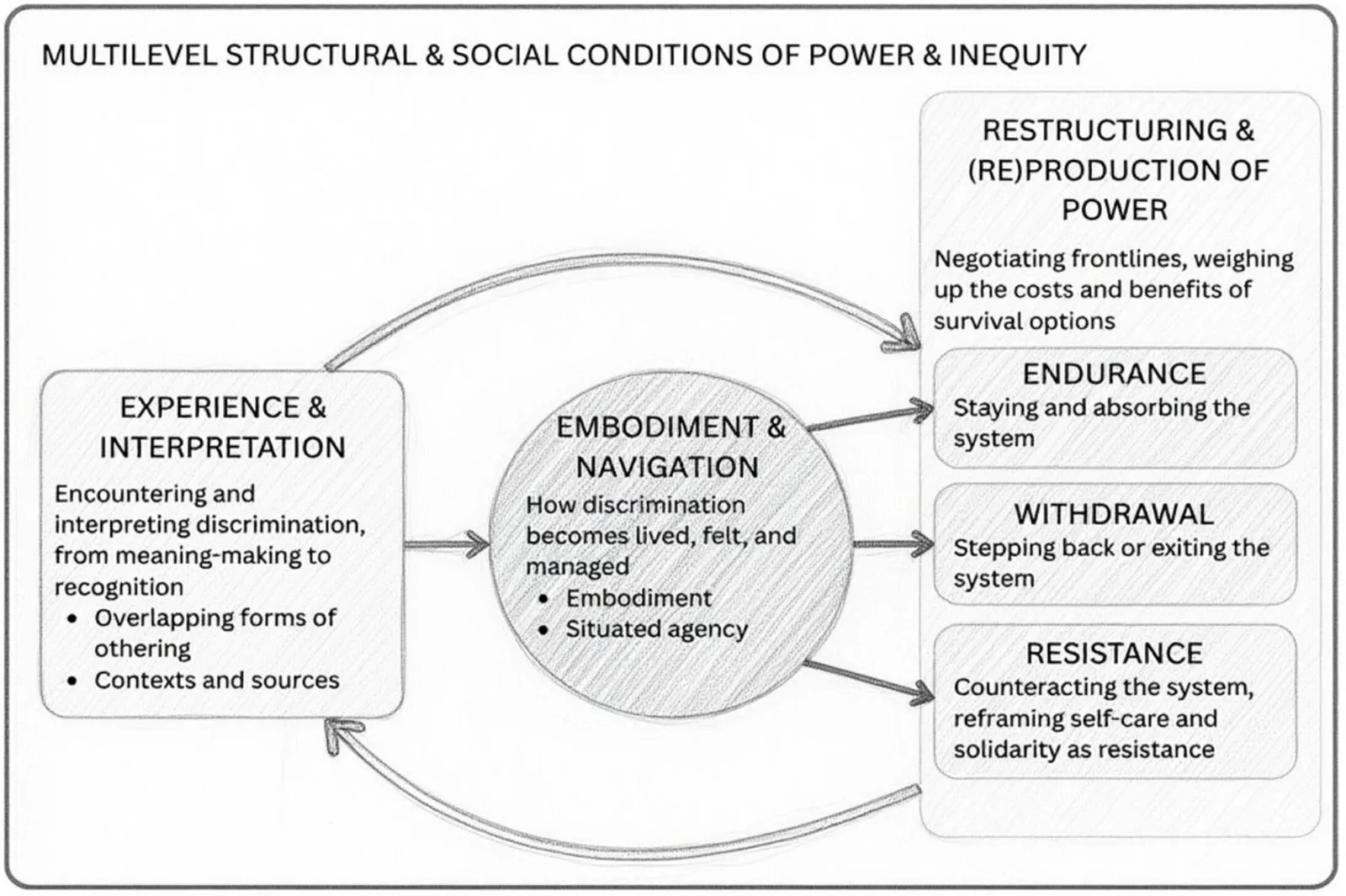
The multilevel discrimination trajectories model (own illustration)

By integrating theories of embodiment [48, 49] with intersectional and affective critiques of institutions [8–10], this study contributes a nuanced framework for understanding discrimination as a process that is simultaneously corporeal, relational, and structurally (re)produced. Building on these insights, we conceptualise student discrimination in higher education as a cyclical, embodied process shaped by multilevel power relations (see Fig. 1). Rather than unfolding in a linear sequence, students continuously move through and may re-experience three interconnected stages when navigating discriminatory environments: (1) encountering and interpreting discrimination; (2) the embodied experience and situated navigation of it; and (3) the (re)structuring and (re)production of power.

Each stage shapes the next, so for example, how a student interprets a discriminatory moment influences how they embody and respond to it. These responses, in turn, influence their future engagement with the institution, either reinforcing existing norms or unsettling them. Within this cyclical process, students adopt different survival strategies when negotiating inequity: endurance, withdrawal, and resistance. These trajectories reflect both individual coping responses and the broader structural and relational conditions under which students survive unequal environments. As previously mentioned, such embodied responses should not be considered as discrete categories, but as rather dynamic and fluid responses that are shaped by social positioning, emotional landscapes, and shifting assessments of risk and possibility [15].

Endurance often meant staying in the system at a personal cost. Students described how they persisted through discriminatory environments by suppressing their emotions, over-performing, and remaining constantly vigilant. These practices echo the minority tax, the invisible labour involved in navigating institutions dominated by institutional whiteness while dealing with misrecognition, threat, and conditional belonging [31]. Such endurance is not merely psychological but actual embodied survival work [30, 60], shaped by fear of retribution, pressure to succeed, and the absence of safe institutional pathways for addressing harm.

Withdrawal emerged as an emotional and tangible strategy for self-preservation. For some students, this meant distancing themselves from certain academic spaces or social groups, while for others it involved contemplating leaving university or even the country altogether. These forms of withdrawal are often misread as disengagement or failure. In our case, it functioned as a form of boundary-setting and a refusal to endure chronic harm, aligning with the idea of withdrawal as an act of self-care in the face of institutional abandonment [50].

Resistance encompassed both overt and quiet forms of critique. For some, this meant speaking up, building solidarities, or engaging in political work. For others, it meant drawing boundaries, resting, or reclaiming joy, forms of quiet resistance that challenge institutional demands. These acts shift the narrative from “resilience” to agency: not coping with harm but confronting its conditions [47, 52]. However, resistance in this context should not be romanticised, as it is often emotionally costly and met with indifference or pushback. Furthermore, resistance is unequally accessible depending on students’ own positioning [50].

The trajectories resulting from our analysis should not be understood as static, as students often moved between different forms of agency, accepting some contexts while resisting others, or withdrawing when resistance became too risky. This fluidity resonates with the accommodation and resistance framework, which conceptualises how marginalised individuals navigate environments that simultaneously offer opportunities and (re)produce inequalities [61–63]. Rather than positioning students as passive recipients of discrimination or as fully autonomous actors capable of overcoming structural barriers through individual effort, the framework highlights how adaptation to and contestation of unequal conditions often coexist in everyday practices [61]. In a similar way, students in our study shifted between endurance, withdrawal, and resistance depending on context, perceived risks, and available resources. Their choices were shaped by the costs and benefits of different responses, revealing the ongoing labour of navigating exclusion [15]. This labour, while invisible to institutions, has tangible effects on wellbeing, academic engagement, and long-term aspirations [31, 60].

### Strengths and limitations

Our study offers several strengths that deepen understanding of discrimination in higher education and its embodied effects among students. Firstly, its subject-oriented, inductive approach prioritised students’ own meaning-making processes, allowing for a rich and nuanced reconstruction of lived experience. Rather than imposing predefined categories, we used an open definition of discrimination, enabling the emergence of under-researched identity axes and context-specific forms of exclusion. This approach also allowed us to trace not just what discrimination is, but how it is embodied, navigated, and resisted over time.

Secondly, our research was grounded in a self-reflexive methodology, with sustained attention to power relations in knowledge production, emotional labour in interviewing, and the ethical complexities of working with potentially marginalised participants on sensitive topics. One of the strengths of this approach, its openness and relational depth, also brings ethical tensions that warrant reflection around responsibility, potential re-traumatisation, and the emotional aftercare of both participants and researchers. The interview space allowed for moments of emotional honesty, resonance, and deep reflection. This strategy aligns with socially critical forms of research that recognise the research encounter as a space of epistemic and affective significance [41]. However, it also demanded attentiveness to the boundaries between research and therapeutic engagement, especially given the institutional voids students often face when naming harm. We do not frame this as a methodological limitation, but rather as a reminder of the emotional responsibility borne by researchers when institutional care is absent. Holding space for vulnerability is not a flaw, but it is not neutral and requires ethical accountability beyond the interview setting.

Limitations include potential language accessibility barriers, which may have shaped who felt able to participate and what could be articulated during interviews. Nevertheless, participants had the option to participate in English. Furthermore, a self-selection bias may be present, as students who were more directly affected by or politicised around issues of discrimination might have been more likely to engage. However, the insecurity some participants expressed, even during the interviews, about whether their experiences constituted discrimination does not support this notion. In addition, all participants were recruited from a major urban centre in Germany. As such, the experiences captured here may not reflect the realities of students studying in smaller cities or rural areas, where institutional dynamics and access to support structures may differ.

These limitations do not diminish the quality of the findings but help contextualise their scope. They highlight the need for further research across varied academic environments and underscore the importance of contextual sensitivity in capturing the complex and situated realities of discrimination. Additionally, our limitations also speak to the broader issue that this study seeks to address, namely that those most affected by structural harm are often also those most burdened with the labour of naming, explaining, and resisting it.

## Conclusion

This study contributes to the growing evidence that discrimination in higher education is not an isolated incident but a multidimensional, embodied process that shapes student mental health and wellbeing. Through a qualitative intersectional, multi-level lens, we show how discriminatory experiences are shaped by institutional norms and power relations, and how students respond through strategies of endurance, withdrawal, and resistance. These responses are not only personal, but political and relational, reflecting the structural conditions under which students must navigate their academic lives. As long as universities continue to frame inequity as an individual issue rather than a systemic one, the burden of recognition and adaptation will remain on those most affected. To promote genuine equity in academic environments, universities must move beyond symbolic commitments and implement transparent accountability mechanisms, intersectional pedagogy, and structural reforms that confront the very norms producing harm through discrimination.

## Supplementary Information

Below is the link to the electronic supplementary material.

Supplementary Material 1

## Data Availability

The analysis and materials used in this manuscript are not publicly available due to privacy restrictions imposed by the institutional ethics board. However, they can be obtained from the corresponding author upon completion of a privacy and fair use agreement.
