# Supplementary material for: “Not only standing for, but standing up for diversity”: an intersectional qualitative analysis of how university students embody and resist discrimination
Source: Int J Equity Health. 2026 Sep 5;25:205. doi: 10.1186/s12939-026-03003-w (PMC13551830; doi:10.1186/s12939-026-03003-w)
Supplement: Supplementary file 1 [file 12939_2026_3003_MOESM1_ESM.pdf]

Appendix A Supplementary material: Interview guide
Titel: “Not only standing for, but standing up for diversity”: An intersectional qualitative analysis of how university students embody and resist discrimination

Interview phase	Leading questions	Follow-up questions	Notes
**1. Introduction and self-identification**	In this interview, we will talk about **discrimination (i.e. disadvantage/unequal treatment) during your studies**. This has a lot to do with **identity (how others see you/how you perceive yourself)**.<br>○ **Tell me, what makes you the person you are?**	○ What **aspects** are **relevant** to your **self-identification**?<br><br>○ Are there particular **roles** in which you feel particularly recognised (e.g. student, father/mother...)?	
**2. Experiences with (multidimensional) discrimination**	Now that we've talked about aspects of your identity, I'd like to talk more about your **specific experiences at university.**<br><br>○ To what extent are there or have there been **situations during your studies** in which you felt **particularly disadvantaged** - i.e. discriminated against?<br>○ … or **particularly supported**?	○ Tell me, **who or what played a role** in these situations?<br>○ To what extent did you have the feeling that this experience was caused by **individuals** or rather by **structural conditions** i.e. your institution?<br>○ To what extent was this a **one-off or repeated experience**?<br>○ Sometimes you only **realise afterwards** that a situation was discriminatory. **What was it like for you?**<br>○ To what extent do you think that such experiences are also linked to **aspects of society** as a whole (i.e. norms, values or political structures)?	
**3. Implications for (mental) health**	I would be interested to know how such experiences have affected you and your life.<br>○ **What do you notice in yourself when you tell me about such experiences?**	○ **How did you feel after these experiences?**<br><br>○ **How have these experiences influence your**…mental health, motivation to study, dropping out, changing universities, physical health, social interactions/relationships with	

Interview phase	Leading questions	Follow-up questions	Notes
	○ **What have these experiences done to you?**	teacher/fellow students or help-seeking behaviour?	
**4. Coping mechanisms**	We have talked about the consequences these experiences have had on you. Now I would like to know how you have dealt/deal with these situations.<br>○ **How has the situation developed since?**<br>○ **How do you feel about it today?**	○ What **helped** you to cope/deal with this?<br>○ What resources at university or outside were helpful?<br>○ What made it more **difficult**?	
**5. University factors and structural conditions**	Finally, I would like to talk to you about the role of the university and possible improvements...<br>○ If not mentioned by itself: **To what extent did you experience support at university?**	○ **What could the university do to support you or people in a similar situation?**<br>○<br>○ Alternative: Wish list: **Imagine the perfect university for you - what would it look like?**	
**6. Conclusion**	Thank you, open topics, rounding up	○ Is there **anything else** you would like to add or emphasise in relation to your experiences of discrimination at university?<br>○ (If not clear from the interview): How did it come about that you wanted to talk with me about this topic/participate in the study?	
